# Plasma Blood Levels of Tafenoquine following a Single Oral Dosage in BALBc Mice with Acute *Babesia microti* Infection That Resulted in Rapid Clearance of Microscopically Detectable Parasitemia

**DOI:** 10.3390/pathogens12091113

**Published:** 2023-08-31

**Authors:** Dana G. Mordue, Synthia J. Hale, William E. Dennis, Chau V. Vuong, Xiu-Min Li, Nan Yang, Gary P. Wormser

**Affiliations:** 1 Department of Pathology, Microbiology and Immunology, New York Medical College, Valhalla, NY 10520, USA; 2 Walter Reed Army Institute of Research, Experimental Therapeutics Branch, Department of Drug Development, Silver Spring, MD 20910, USA; william.e.dennis.civ@mail.mil (W.E.D.);; 3 General Nutraceutical Technology, LLC, Elmsford, NY 10523, USA; nan.yang@gnt-us.com; 4 Division of Infectious Diseases, New York Medical College, Valhalla, NY 10520, USA; gwormser@nymc.edu

**Keywords:** babesiosis, treatment, *Babesia microti*, tafenoquine, blood levels

## Abstract

Previous studies of mice infected with *Babesia microti* have shown that a single dose of tafenoquine administered orally is extremely effective at decreasing microscopically detectable parasitemia. However, a critical limitation of studies to date is the lack of data concerning the plasma levels of tafenoquine that are needed to treat babesiosis. In the current study, we begin to address this gap by examining the plasma levels of tafenoquine associated with the rapid reduction of *B. microti* patent parasitemia in a mouse model of babesiosis. In the current study, we infected BALB/c mice with 1 × 10^7^ *B. microti*-infected red blood cells. Two days post-infection, mice were treated with 20 mg/kg of tafenoquine succinate or vehicle control administered orally by gavage. Parasitemia and plasma levels of tafenoquine were evaluated every 24 h post-treatment for 96 h. This allowed us to correlate blood plasma levels of tafenoquine with reductions in parasitemia in treated mice. Consistent with previous studies, a single oral dose of 20 mg/kg tafenoquine resulted in a rapid reduction in parasitemia. Plasma levels of tafenoquine 24 h post-administration ranged from 347 to 503 ng/mL and declined thereafter. This blood plasma tafenoquine level is similar to that achieved in humans using the current FDA-approved dose for the prevention of malaria.

## 1. Introduction

Babesiosis is a potentially life-threatening disease caused by apicomplexan piroplasm parasites that infect red blood cells (RBCs). In the United States (US), babesiosis is primarily caused by *Babesia microti.* Other babesia organisms of human or veterinary importance in North America include, but are not limited to, *Babesia duncani*, *Babesia odocoilei*, *Babesia bovis*, and *Babesia bigemina.* At this point, clarification is still needed regarding the various *Babesia* species that affect humans worldwide. *B. divergens* and *B. venatorum* are of particular importance in both Europe and Asia [1,2,3,4,5,6,7]. Babesiosis is also a significant cause of morbidity and mortality in veterinary medicine. More effective treatments for both human and zoonotic babesiosis are needed.

*B. microti* parasites are transmitted by *Ixodes scapularis* ticks. Transmission by transfusion of contaminated blood also occurs as the parasite survives blood-banking procedures [1,8,9]. Clinical features of babesiosis range from asymptomatic infection or mild disease to severe, life-threatening illness [10]. The recommended treatment for symptomatic babesiosis is 7–10 days of combination therapy with either azithromycin plus atovaquone or clindamycin plus quinine [5,11]. Currently recommended therapies, however, do not rapidly decrease parasitemia; thus, RBC exchange transfusion is often a consideration in individuals with a high level of parasitemia [5,12,13,14,15,16]. Highly immunocompromised patients with babesiosis typically require at least six weeks of treatment [13,16]. In patients treated with the immunosuppressive monoclonal antibody, rituximab, *Babesia* infection can persist for more than 2 years despite long-term antiparasitic drug therapy [16]. Immunocompromised individuals often have severe and prolonged bouts of *Babesia* infection, even with the most effective current treatments. In addition, *Babesia* parasites that are resistant to atovaquone alone or atovaquone and azithromycin may emerge [17,18,19]. More effective treatment options for babesiosis, particularly for immunocompromised individuals or for those with babesiosis refractory to current therapies, need to be developed.

Tafenoquine (originally designated compound WR 238605) is an 8-aminoquinoline that was discovered by scientists at the Experimental Therapeutics branch of the Walter Reed Army Institute of Research (WRAIR). In 2018, Tafenoquine was approved by the United States Food and Drug Administration (FDA) to prevent malaria and eradicate the hepatic stage of the relevant *Plasmodium* species [20,21,22]. It is effective for both the blood and liver stages of the parasite [23]. An important characteristic of tafenoquine is its long half-life in humans. Unlike primaquine, tafenoquine is eliminated slowly and has an estimated terminal half-life in blood of 14 days compared to 6 h for primaquine. Like primaquine, it is contra-indicated for individuals with glucose-6-phosphate dehydrogenase (G6PD) deficiency and requires quantification of G6PD prior to prescribing. The exact mechanism of action, effective metabolites, and molecular target of tafenoquine for *Plasmodium* is unknown, particularly for blood-stage parasites. However, tafenoquine has been shown to interfere with mitochondrial function, cause the production of reactive oxygen intermediates, and induce apoptotic-like cell death in diverse protozoa, including *Plasmodium* [22,24]. Although tafenoquine has been approved for the radical cure of *P. vivax* in humans, there is increasing evidence that optimal dosing may be impacted by both human and parasite genetics [25]. Additional clinical trials have been initiated to determine whether increasing the therapeutic dose will impact the toxicity and effectiveness for treatment of *Plasmodium vivax* (Clinical Trials.gov Southeast Asia Dose Optimization of Tafenoquine SEADOT; Escalating Monthly Doses of Tafenoquine in Healthy Volunteers TQ).

The first study that examined the efficacy of tafenoquine as a potential treatment for babesiosis was carried out by WRAIR in 1997. The compound WR248605 (tafenoquine) was found to be much more effective than clindamycin and quinine in a hamster model of babesiosis caused by the Gray strain of *B. microti* (passaged in hamsters) [26,27]. In the WRAIR study, the compound was delivered intramuscularly to hamsters at a dose of 3.25 mg/kg twice per day for 4 days and resulted in rapid elimination of patent parasitemia within three days with no parasite recrudescence. Pharmacokinetic studies to evaluate blood or plasma levels associated with the elimination of parasites were not performed. In 2019, we revisited the potential use of tafenoquine as a therapy for babesiosis but used severe combined immune deficiency mice (SCID) instead of immune-competent hamsters. We showed that a single oral dose of tafenoquine of 20 mg/kg was capable of rapidly decreasing *B. microti* parasitemia below detectable levels in blood smears of SCID mice, which lasted for nearly a month post-treatment [28]. A single oral dose of tafenoquine achieved a >90% reduction in the level of parasitemia within 4 days of initiating treatment, even in mice with pretreatment levels of parasitemia that exceeded 20%. Although there are only limited data on humans in response to standard therapies, combination therapies have shown only a 4.1–12.9% reduction per day in the DNA copy numbers of *B. microti* [19]. Since our study, tafenoquine has been shown to be highly effective against not only *B. microti* but other *Babesia species* as well. Studies by M. Liu et al. [29] in 2021 showed that a single oral dose of 20 mg/kg of tafenoquine resulted in a rapid reduction in *B. rodhaini* parasitemia in immune competent and in SCID mice [29]. In the same publication, they also showed that treatment with a single dose of tafenoquine resulted in rapid suppression of *B. gibsoni* in dogs that were splenectomized [29]. Similar to our study, tafenoquine treatment resulted in a distinct parasite phenotype post-treatment immediately prior to reduction of parasites below the level of detection by microscopy in stained blood smears. Their study showed that the change in parasite phenotype in mice treated with tafenoquine correlated with the increased expression of parasite genes associated with resistance to oxidative stress. Like our study with *B. microti*, single dose tafenoquine treatment was not sufficient prevent parasite recrudescence in either BALB/c mice or SCID mice or in splenectomized dogs.

Since the publication of our original findings, there have been three published case reports in which patients with babesiosis refractory to standard treatment were treated with tafenoquine [30,31,32]. In each case, a 600 mg loading dose of tafenoquine was administered over a 3-day period, followed thereafter by either a 200 mg or 300 mg dose administered once weekly for at least 6 weeks. While tafenoquine treatment was associated with a marked decrease/suppression in parasitemia, when the tafenoquine was discontinued, parasite recrudescence was evident in at least one of the cases.

Although tafenoquine has been shown to be highly effective against multiple *Babesia species* in a variety of animal models, plasma blood levels of tafenoquine required for effective therapeutic activity against *Babesia* species have not been determined. The blood plasma levels of tafenoquine that need to be achieved and the length of time such drug levels need to be maintained to be effective for the treatment of babesiosis are unknown. Our current study is a first step toward addressing this knowledge gap to help inform appropriate tafenoquine dosing in humans. In the current study, we determined the tafenoquine blood plasma levels, in combination with measuring parasitemia, before treatment with a single dose of 20 mg/kg tafenoquine and after treatment at 24 h intervals for 96 h in mice with babesiosis.

## 2. Materials and Methods

### 2.1. Infection and Parasitemia

Mice throughout the study were housed in the New York Medical College Laboratory Animal Complex in Animal Biosafety Level 2 conditions. The study protocol, “Tafenoquine as a Potential Revolutionary Treatment for Babesiosis”. (Protocol 13958 Principle Investigator D. Mordue) was approved by the New York Medical College Institutional Animal Care and Use Committee (IACUC). Female BALB/c mice (n = 45) 7 weeks of age were obtained from Jackson Laboratories. *Babesia microti* was obtained from the American Type Culture Collection (ATCC 30221 Gray strain isolated from a patient [33]). Although tafenoquine has been shown to be effective against a broad range of *Babesia* species, this study used the Gray strain to be consistent with our previous study of tafenoquine as a treatment for babesiosis in immune-deficient SCID mice [28]. Extended in vitro passage conditions for *B. microti* remain unknown, and passage is limited primarily to serial propagation in hamsters or mice followed by freezing of infected RBCs in liquid nitrogen. Infected RBCs were thawed from storage in liquid nitrogen and immediately injected intraperitoneally (IP) in BALB/c mice. Total RBCs were isolated from the blood of the infected mice 5–7 days post-infection and used to infect 10 additional BALB/c mice IP. Female BALB/c mice were infected with 100 μL of blood intraperitoneally that was transferred from these *B. microti*-infected BALB/c mice five days post-infection when parasitemia levels were 20–30% (approximately 1 × 10^7^ *B. microti*-infected red blood cells). Parasitemia in mice was monitored every 24 h using standard methods to collect a drop of blood by tail snip to create a blood smear on a slide. Blood smears were fixed with 100% ice-cold methanol for 5 min, followed by Giemsa stain for 30 min. Blood smears were examined by bright field microscopy using a 100× oil objective. To quantify the level of parasitemia, the mean of at least 3 counts of 100 RBCs was determined.

### 2.2. Tafenoquine Treatment

Twenty *B. microti*-infected mice were treated with tafenoquine succinate salt (Sigma Immunochemicals) (20 mg/kg/15.9 mg/kg free base) or vehicle control (n = 5 mice) by oral gavage on day two post-infection when patent parasitemia levels were between 5–10%. Tafenoquine was resuspended with 0.5% *w*/*v* hydroxyethyl cellulose and 0.2% *v*/*v* Tween-80 immediately prior to use, as previously described [34].

### 2.3. Blood Collection

At 0, 24, 48, 72, and 96 h post-treatment, 4–5 mice per time point were anesthetized. A single drop of blood was collected from anesthetized mice using the tail snip procedure, blood was deposited on a microscope slide, and a thin blood smear was created for fixation and staining with Giemsa to monitor parasitemia by bright field microscopy. While mice were anesthetized, blood was collected from the heart using a 1 mL tuberculin syringe in order to collect approximately 1 mL of blood. Tubes for blood collection contained 3% buffered sodium citrate to prevent blood clotting. Plasma was isolated by centrifugation at 1000× *g* for 10 min using a refrigerated microfuge to pellet cells. Parasitemia levels and tafenoquine plasma blood levels were from the same groups of mice.

### 2.4. Plasma Tafenoquine Levels

Plasma samples were stored at −80 °C until ready for analysis. 20 µL of 0.5% *w*/*v* zinc sulfate in water was added to 100 µL of plasma. The sample was vortexed briefly and extracted with 240 µL of 90% acetonitrile; 10% methanol containing mefloquine was used as an internal standard. The sample was vortexed vigorously for 10–15 s, then centrifuged at 13,000 rpm for 10 min at 4 °C. The supernatant was transferred to an autosampler vial for analysis. Calibration curves and QC samples were prepared by adding standard solutions into blank BALB/c mouse plasma and extracted in the same manner. Tafenoquine reference standard from the WRAIR depository was used to prepare the standard solutions. Blank BALB/c mouse plasma was purchased from BioIVT (Westbury, NY, USA).

Tafenoquine and the mefloquine internal standard were detected by multiple reaction monitoring (MRM) on a Waters TQ-s-Micro mass spectrometer using electrospray ionization (ESI) in positive ion mode, monitoring the *m/z* transitions. The settings for the ESI source were as follows: capillary voltage 0.5 kV, cone voltage 50 V, desolvation temperature 500 °C, desolvation gas 1000 L/h, and cone gas 10 L/h. Liquid chromatography separation was performed on a Waters ACQUITY UPLC and a CORTECS C18, 2.7 µm, 21 × 50 mm column. The mobile phase was 0.1% formic acid in water (A pump) and 0.1% formic acid in acetonitrile (B pump). Moreover, 2 µL of each plasma sample was injected and separated using a linear gradient with a flow rate of 0.450 mL/min, ramping from 5% to 95% B (acetonitrile) from 1 to 2.50 min. This solvent composition was maintained for 4.50 min. The solvent composition was returned to 5% B (acetonitrile) at 4.51 min, and the run stopped at 5.75 min. The plasma half-life of tafenoquine was estimated using GraphPad Prism.

### 2.5. Microscopic Analysis of Parasitemia

A thin blood smear from each mouse was fixed for 10 min in ice-cold methanol. Fixed blood smears were stained with Giemsa staining solution for 30 min at room temperature, rinsed with water, and allowed to dry. Stained slides were viewed under a 100× oil objective using bright field microscopy to quantify parasitemia. The average level of parasitemia and standard deviation per mouse per time point was determined by calculating the number of infected RBCs per 100 total RBCs using three independent counts.

### 2.6. Statistical Analysis

Statistical analyses for parasitemia levels in tafenoquine-treated and untreated mice per day post-treatment were calculated in GraphPad Prism 9 software using 2-sample multiple-paired *t*-tests. *p*-values less than 0.05 were considered significant.

## 3. Results

**Figure 1:**
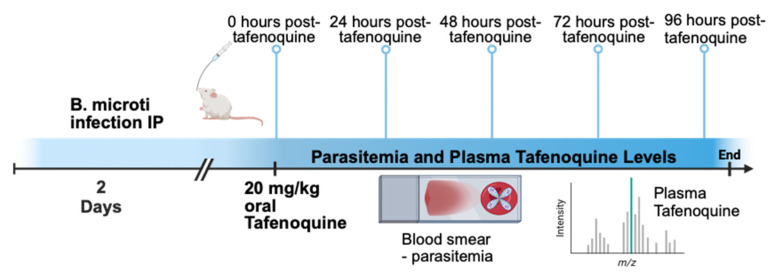
Experimental model for tafenoquine treatment of babesiosis. Mice were infected with *B. microti*. Two days post-infection, mice were treated with 20 mg/kg tafenoquine by oral gavage. Blood was collected from mice 0, 24, 48, 72, and 96 h after tafenoquine treatment to monitor parasitemia by thin blood smear and plasma tafenoquine levels by liquid chromatography-tandem mass spectrometry.

**Figure 2:**
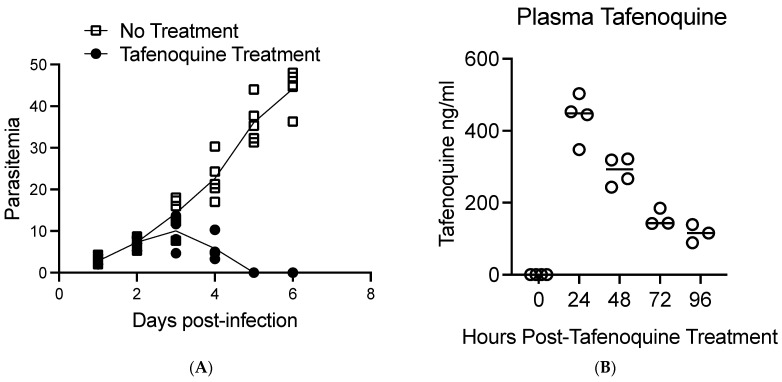
Comparison of tafenoquine plasma levels and parasitemia in mice treated orally with 20 mg/kg of tafenoquine. (**A**). Parasitemia before and after oral treatment with tafenoquine or vehicle alone on day 2 post-infection. Parasitemia was determined by bright field analysis of Giemsa-stained blood smears. Three counts of 100 red blood cells were performed at each time point for each mouse. (**B**). Plasma levels of tafenoquine post-treatment. Plasma levels of tafenoquine were determined by high-performance liquid chromatography-tandem mass spectrometry. Tafenoquine was administered on day two post-infection. Therefore, the 24 h time point post-tafenoquine treatment in (**B**) corresponds to the parasitemia levels shown in (**A**) on day three post-infection. Values for each individual mouse are shown along with a line that marks the average value for the mice assessed. Three to five mice were used per group and time zero represents control mice that did not receive tafenoquine.

Figure 1 shows a diagram of the overall approach to the study. BALB/c mice were infected with approximately 1 × 10^7^ *B. microti*-infected red blood cells intraperitoneally (IP), and parasitemia was monitored by brightfield microscopy of Giemsa-stained blood smear daily. On day 2 post-infection, when parasitemia was approximately 5–10%, mice were treated with 20 mg/kg of tafenoquine by oral gavage or vehicle alone. At 0, 24, 48, 72, and 96 h post-treatment, 4–5 mice per time point were anesthetized, and blood was collected to monitor parasitemia by blood smear and blood plasma tafenoquine by liquid chromatography-tandem mass spectrometry. As shown in Figure 2A, administration of 20 mg/kg of tafenoquine orally resulted in significant suppression of parasitemia within 48 h of treatment. The mean parasitemia levels in mice that received vehicle alone was 23% (range: 17–30%), compared to less than 6% (range: 3–10%) in tafenoquine-treated mice by 48 h post-treatment (*p* < 0.005). Tafenoquine treatment was associated with both a reduction in the level of parasitemia (Figure 2A) and with the development of an aberrant parasite phenotype, visible within 48 h of treatment and characterized by a ghost-like appearance with only faint chromatin staining, as we and others have previously described [28,29]. The high level of effectiveness of a single oral dose of 20 mg/kg of tafenoquine to suppress *B. microti* parasitemia to levels undetectable on Giemsa-stained blood smears by day 5 post-infection, i.e., 3 days after being treated with tafenoquine, is consistent with our previous study of the same oral dose of tafenoquine administered to SCID mice infected with *B.* *microti* [28].

Figure 2B shows the plasma levels of tafenoquine per mouse 24, 48, 72, and 96 h post-treatment. Plasma levels of tafenoquine were 437 ng/mL on average (range 347 ng/mL–503 ng/mL) 24 h post-delivery of oral tafenoquine. Plasma levels of tafenoquine were reduced to an average value of 288 ng/mL (range 243 ng/mL–322 ng/mL) 48 h post-treatment, to an average value of 157 ng/mL (range 142 ng/mL–185 ng/mL 72 h post-treatment and to an average value of 114 ng/mL (range 88 ng/mL–139 ng/mL) at 96 h post single-dose treatment. The elimination half-life of tafenoquine in our study is estimated to be approximately 37.9 h in mice with babesiosis.

Thus, our results show that a 24 h post dosage level of 347 to 503 ng/mL of tafenoquine correlated with a rapid reduction of *B. microti* parasitemia in infected BALB/c mice. Of note, our data show that 347 ng/mL of tafenoquine at 24 h post-treatment correlated with a decrease in parasitemia to below the level of detection in blood smears within 72 h.

## 4. Discussion

To date, a limitation of all studies of tafenoquine as a treatment for babesiosis in animal systems is that blood levels of tafenoquine were not determined; the impact of active babesiosis on tafenoquine absorption and excretion in animals has never been assessed. Thus, the goal of the current study was to begin to provide information about tafenoquine plasma levels at various time points after administration of a single oral dose to *B. microti*-infected mice and determine whether the drug levels achieved correlated with effective suppression of *B. microti* parasitemia. This information is critical in order to determine whether the tafenoquine doses approved by the FDA for the prevention of malaria are likely to be effective against babesiosis in humans.

The results of the current study show that a 24-h post dosage level of 347.6 to 503 ng/mL of tafenoquine correlated with a rapid reduction of *B. microti* parasitemia in infected BALB/c mice. While we observed peak plasma levels of tafenoquine at 24 h, based on previous studies, it is likely that the peak plasma level of tafenoquine after an oral challenge was reached prior to our 24 h time-point [29]. The estimated elimination half-life of plasma tafenoquine in our study was approximately 37.9 h, which is slightly shorter than in previous studies in mice that showed an estimated half-life of 50–60 h [29,34]. The plasma half-life of tafenoquine in mice is known to be substantially shorter than in humans, where it is estimated to be approximately 14 days. Of note, the lowest effective blood level at the 24 h time point was not determined in this study. In addition, the tafenoquine plasma levels rapidly fell to an average value of 288 ng/mL 48 h post-treatment, to 157 ng/mL 72 h post-treatment, and 114 ng/mL at 96 h post single-dose treatment. Although recrudescence of parasitemia was not determined in the current study, our previous publication showed that a single oral dose of 20 mg/kg of tafenoquine suppressed recrudescence as determined by microscopy for greater than 20 days [28]. Although peak blood levels were not determined in this study, plasma levels that exceed 300 ng/mL are clearly achievable in humans who receive the FDA-approved loading dose of 600 mg of tafenoquine administered over 3 days (200 mg/day) followed by 200 mg once weekly [35,36]. For example, in one large human study, the mean plasma tafenoquine concentrations at the multiple time points assessed between weeks 4 and 52 ranged from 314.7 ng/mL to 354.7 ng/mL, and the maximum tafenoquine concentration documented was 877.7 ng/mL [35].

A limitation of our study is that it does not provide a dose titration response to determine the effectiveness of lower doses of tafenoquine or provide data on how long a specific tafenoquine plasma level needs to be sustained in order to treat or potentially eradicate the parasite. However, in a previous study, we did show that oral delivery of 5 mg/kg of tafenoquine (i.e., one-quarter of the dose used in this study) had no demonstrable efficacy in suppressing *B. microti* parasitemia in SCID mice [28]. Thus, it is likely that the effective dose for tafenoquine for babesiosis will be similar to the dose approved by the FDA for the prevention of malaria. Our study also did not evaluate whether *B. microti* infection had an impact on plasma tafenoquine levels, as plasma levels were not measured in the absence of infection.

In conclusion, our current study suggests, but does not unequivocally prove, that the tafenoquine dosing regimen that is used to prevent malaria and has been well studied for safety and efficacy is also likely to provide adequate drug levels to treat *B. microti* parasitemia. Peak blood levels with this regimen in humans can be expected to typically exceed 300 ng/mL [35,36].

## 5. Important Areas for Future Research

The current study is the first to examine plasma levels of tafenoquine associated with a rapid reduction of *B. microti* parasitemia. Further studies that combine tafenoquine blood and plasma pharmacokinetic analysis with parasite reduction and clearance are necessary to establish therapeutic tafenoquine doses, not only for *B. microti* species, including those resistant to current treatments such as atovaquone and azithromycin but also for other *Babesia* that cause human babesiosis including *B. duncani*, which appears to be more resistant than *B. microti* to at least some treatments [5,17,19,31,37,38]. These studies are needed to establish the blood/plasma levels of tafenoquine necessary to kill parasites, as well as to determine how long the effective blood/plasma levels need to be maintained. It is also important to evaluate whether a therapeutic dose of tafenoquine can be established that is capable of not only a rapid reduction of parasitemia but also of complete clearance of parasites to prevent recrudescence. Our previous study that used a single oral dose of 20 mg/kg of tafenoquine to treat immune-deficient SCID mice with patent *B. microti* infection reduced parasitemia to levels not detectable by microscopy for nearly a month, but recrudescence was not prevented. Importantly, however, the recrudescent parasites had not developed resistance to tafenoquine, as they remained susceptible to the drug [28]. It will also be important to evaluate the effectiveness of tafenoquine in combination with other current therapies in use for human babesiosis, as well as with potential emerging therapies [5,39,40]. For example, a recent study showed that tafenoquine treatment alone, or combination therapy of tafenoquine and artesunate, were both highly effective in their ability to reduce *B. microti* parasitemia in mice, but only the combination therapy prevented parasite recrudescence [41].

## Data Availability

The authors confirm that the data supporting the findings of this study are available within the article. However, the data that support the findings of this study are also available from the corresponding author [D.G.M.] upon reasonable request.
